# Evaluation of volumetric wear loss and pain scores of the digitally and conventionally manufactured occlusal splints for individuals with sleep bruxism

**DOI:** 10.1590/1678-7757-2025-0052

**Published:** 2025-03-31

**Authors:** Sevda Miray Soydaş Smail, Zeliha Şanivar Abbasgholizadeh, Erkut Kahramanoğlu

**Affiliations:** Marmara University Faculty of Dentistry Department of Prosthodontics Istanbul Turkey Marmara University Faculty of Dentistry, Department of Prosthodontics, Istanbul, Turkey

**Keywords:** Sleep bruxism, Occlusal splint, Volumetric loss

## Abstract

**Introduction:**

According to the latest international consensus in 2018, sleep bruxism is the activity of the masticatory muscles during sleep characterized by rhythmic or non-rhythmic teeth clenching or grinding. Regarding its harmful effects, bruxism is considered one of the predisposing factors of tooth wear and temporomandibular joint diseases. Occlusal splint therapy is the most frequently used treatment for minimizing these harmful effects.

**Objectives:**

This study compared the volumetric wear loss and pain scores between digitally and conventionally manufactured occlusal splints for individuals with sleep bruxism.

**Methodology:**

A total of 30 individuals diagnosed with sleep bruxism were selected following the inclusion criteria and randomly divided into two groups. Pain scores were subjectively reported using a visual analog scale. Volumetric wear loss of the occlusal splint surface was measured using the Geomagic software. Data were analyzed with SPSS version 25.0.

**Results:**

At the six-month follow-up, conventionally manufactured splints (103.53±41.23) showed a volumetric loss significantly higher than that the digital ones (62.33±26.29) (p=0.005). We found no significant difference between the two splint types regarding VAS scores.

**Conclusion:**

Occlusal splint wear can gradually alter the balance of occlusal contact and potentially reduce its therapeutic effectiveness, highlighting the importance of using wear-resistant materials. Our findings indicate that digital manufacturing processes provide advantages due to their long-term clinical outcomes.

## Introduction

Sleep bruxism is characterized by masticatory muscle activity during sleep which can be either rhythmic (phasic) or non-rhythmic (tonic). According to the latest consensus on bruxism in 2018, the condition is not classified as a movement disorder nor as a sleep disorder in otherwise healthy individuals.^1 , 2^ In adults, the prevalence of sleep bruxism ranges from 8% to 15%, whereas awake bruxism has a prevalence of 22% to 30%.^3 , 4^

Latest advancements in bruxism classification includes the development of the Standardised Tool for the Assessment of Bruxism (STAB). Introduced in 2023, STAB provides a thorough, multidimensional instrument to assess bruxism by looking at its status, possible effects, causes, and other underlying conditions. It comprises two axes: Axis A and Axis B. Axis A includes a subject-based report containing self-reported data on bruxism state and potential consequences, with an examiner report and an instrumental report. Axis B contains self-reported data about circumstances and factors potentially associated with or causative of bruxism.^5^

Occlusal splint therapy is the most common treatment for reducing the harmful effects of bruxism,^6 , 7^ and one of the most effective treatments for painful temporomandibular disorders.^8^

Occlusal splints can be manufactured from hard or soft materials, but Okeson^6^ (2019) reports that hard occlusal splints have better effects on reducing pain intensity and symptoms of muscle tenderness.

Conventional manufacturing methods for occlusal splints include injection molding and vacuum forming of vinyl acrylic. Rigid occlusal splints manufactured via vacuum forming are completed by adding PMMA (polymethylmethacrylate), which polymerizes spontaneously or by heat, on a plaster model obtained from patients’ intraoral impression. As this method requires technical accuracy and long chair side adjustments to provide passive fitting and proper occlusal contacts on the splint, it has become an uncomfortable method for patients. Porosity formation, polymerization shrinkage, and excessive residual monomer content are critical aspects that affect the structural quality of occlusal splints. These factors are disadvantages in conventional manufacturing, decreasing treatment efficacy.^9 , 10^

Currently, prosthetic restorations are frequently manufactured using computer-aided systems. Computer-aided design (CAD) and computer-aided manufacturing (CAM) eliminate individual mistakes that can occur in conventional processes due to technical precision. In addition to high material quality, they allow for adjusting the restorations in a timely manner. Digital manufacturing processes provide similar advantages in occlusal splint fabrication, thus making it more popular.^11^

Computer-aided manufacturing of occlusal splints employs two distinct technologies: subtractive manufacturing and additive manufacturing. Multiple materials can be used in these production processes, including polycarbonate, polyetheretherketone, and PMMA.^12^

Even with limited splint usage, these materials typically degrade with time in cases of bruxism or parafunction. Wear loss decreases splint durability by affecting the equilibrium of occlusal contacts which is essential for effective splint therapy. Consequently, these impacts adversely affect patient compliance and treatment outcomes both of which are crucial for treatment effectiveness, underlining the necessity for superior quality materials.^13^

This study assessed patients’ pain scores and wear loss rates of occlusal splints fabricated digitally and conventionally. Our null hypothesis posits no difference in both wear loss rates and pain scores between the two methods.

## Methodology

This research was conducted at Marmara University, Department of Prosthodontics. Ethical approval was obtained by Marmara University Clinical Research Council's Ethics committee no. 2022-85 and approval E-68869993-000-1082912, Republic of Turkey, Ministry of Health, Turkish Medicines and Medical Devices Agency.

Based on data from a previous study by Wang, et al.^14^ (2020), a sample size of 30 participants was calculated using G*Power analysis (power 0.95, a=0.05).

Participant allocation was randomized using randomization software (QuickCalcs; GraphPad Software Inc., La Jolla, CA, USA) to ensure unbiased group distribution. A list of participant IDs was entered into the Random Number Generator, shuffled, and sequentially assigned to treatment groups.

Participants underwent impression-taking and fabrication of stabilizing splints for their upper jaws using conventional methods in one group and digital manufacturing in the other.

### Volunteer Selection

Our sample included volunteer individuals who visited Marmara University Faculty of Dentistry, Department of Prosthodontics, with complaints of teeth clenching and grinding. A total of 30 volunteers aged 18-60 years with natural dentition and no temporomandibular disc displacement and degenerative joint diseases (assessed by DC/TMD questionnaires) diagnosed with bruxism using STAB Axis A subject-based and clinically based assessments for self-reported information and clinical evaluations participated in the study. Bruxism-related pain was assessed by Visual Analog Scale (VAS). Exclusion criteria included pregnancy, alcohol or drug addiction, multiple missing teeth in one arch area, TMJ disc disorders, arthralgia, arthritis, adhesions, ankylosis, joint diseases, congenital disorders, myalgia and myofascial pain according to DC/TMD questionnaires.

Prior to stabilization splint application, the diagnosed volunteers received information regarding the anatomy, function, bruxism, and specific application of the splint. Participants were given both written and verbal information regarding the treatment duration, other treatment options, potential discomfort associated with splint application, and its potential advantages. All participants provided written consent.

### Conventional Manufacturing Process

Maxilla impression was taken with a perforated impression tray using alginate impression material (Hydrogum, Zhermack (Dentsply Sirona Dental GmBH, Salzburg, Austria)) to obtain stone (type III) models. 3 mm thick hard vinyl resin material (Pro-form vacuum-forming splint, Keystone Industries, Holland) was pressed onto the stone model with a Plastvac P7 vacuum adapter (Bio-art, Brazil). Splint application to patients’ maxilla was performed by the same dentist according to Okeson^6^ (2019) and canine guidance was provided in lateral movements. Based on this guideline, a hard vinyl acrylic splint material (Keystone Industries, New Jersey, America) was vacuumed on a plaster model. After leveling the splint borders, a small amount of PMMA-based autopolymerizing orthodontic acrylic resin (Imıcryl O-80, Turkey) was added to the anterior portion of the splint as a stop for the lower incisor. After determining the vertical dimension and centric relation (CR) position with this anterior jig, acrylic resin was added to the occluding surface. All occluding areas except for contact on the anterior stop were covered. A small amount of additional acrylic was placed labial to the canine regions to aid future guidance. In the occlusal surface area, teeth tubercle marks were adjusted by preserving the painted contact points on acrylic according to the bite obtained in the centric relation. For the anterior position, the canine ramp was adjusted to allow continuous sliding canine movement during protrusive and laterotrusive movements and to ensure posterior dislocation. After finishing the adjusting procedures, polishing was performed completing the manufacturing of the occlusal splint.

### Digital Manufacturing Process

Computer-aided impressions (CAI) were taken by a 3Shape Trios 3 intraoral scanner (3Shape Dental Systems, Copenhagen, Denmark), and centric relations were scanned in a determined vertical dimension with an anterior jig. Anterior jig was adjusted until the lips were in closed position, and 3 mm distance was provided between maxillary and mandibular arches. Scanned STL data were imported into 3Shape Splint Studio Design Program (3Shape Dental Systems, Copenhagen, Denmark). During bite configuration, the jaws were accurately aligned in the articulator using control points. Following the setting of the insertion direction, the processes for undercut removal and wax trimming were organized and a splint shape was drawn. Splint thickness was set at 3 mm, and a ramp was chosen to elevate the vertical dimension. Occlusal contacts were adjusted through distance maps which showed the potential contacts that would emerge in the program. Finally, an anterior ramp was established to provide canine guidance and occlusal contacts were adjusted by the program's articulation area and adapted. Following the final confirmation of occlusal contacts via virtual articulator, the STL file for the designed splint was exported to a specified folder, and the stabilization splint was fabricated using an Envisiontec D4K Pro 3D printer (Envisiontec D4K Pro Industrial, Gladbeck, Germany) with Envisiontec e-guard resin. Stabilization of the manufactured splint was finalized following the washing and curing procedures.

### Wear Loss Measurement

Each volunteer's occlusal surfaces of the stabilizing splints were scanned thrice using the 3Shape Trios 3 scanning technology (3Shape Dental Systems, Copenhagen, Denmark): at baseline, and at 3 and 6 months post-usage. Scannings were coded by one researcher, and wear loss measurement was conducted using the Geomagic Control 2014.4.0 program by another researcher, preventing any potential bias in wear loss assessment.

**Figure 1:**
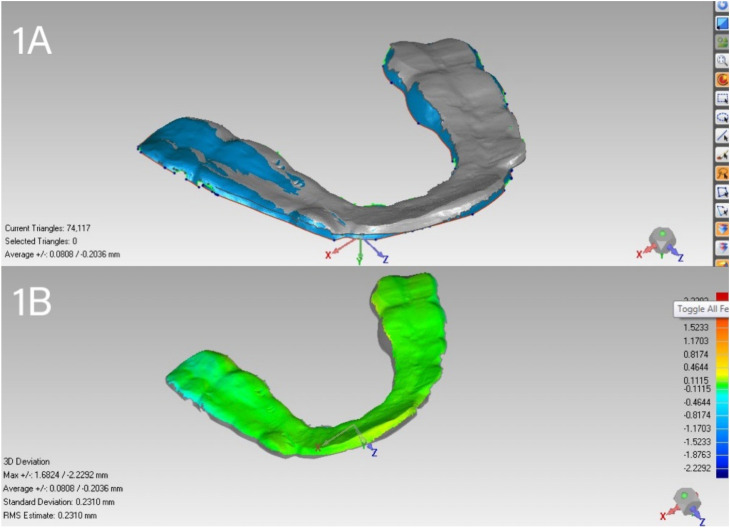
A) Optimal alignment by superimposing occlusal splint scans. B) Color maps of deviation before and after splint wear loss. The green area indicates a deviation of less than 0.05 mm.

Scanning information from the third month was classified as the first test group, whereas scanning data from the sixth month was classified as the second, with baseline scanning data serving as the reference. The algorithm utilized optimal alignment by superimposing the scans between groupings ( Figure 1A ). A three-dimensional examination of the aligned images was conducted to ascertain wear loss. A surface color map was generated to illustrate the exact position and extent of splint surface alterations ( Figure 1B ). Wear volume difference between the two scans was computed and analyzed based on the program's results.

### Patient Subjective Evaluation

Volunteers were requested to assess their pain levels monthly using a visual analog scale (VAS) ranging from 1 to 10. Pain score shows increasing values towards 10 and decreasing values towards 0. We presented the findings categorized by months and groups.

### Statistical Analysis

Data were analyzed using SPSS version 25.0 software. Distribution normality of the variables was assessed using histogram graphs and the Kolmogorov-Smirnov testing. Descriptive analyses were presented using mean, standard deviation, median, and minimum-maximum values. Mann-Whitney's U test was applied to compare non-normally distributed (nonparametric) variables between groups. Changes between groups were analyzed using the Repeated Measures Analysis. P-values below 0.05 were considered statistically significant within the 95% confidence interval (CI).

## Results

**Figure 2:**
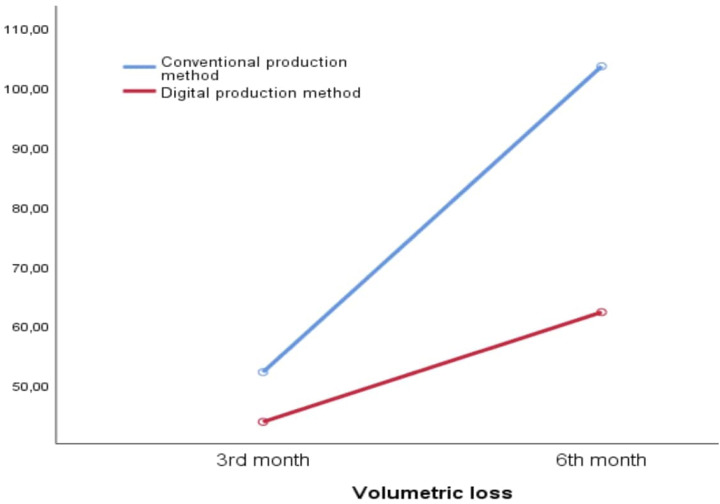
Line graph showing the volumetric loss of occlusal splints at the 3rd and 6th months

A total of 10 male (mean age 31.5) and 20 female (mean age 31.95) volunteers from the Turkish population with a mean age of 31.8 years were included in the study. In the conventional manufacturing process, the average volumetric loss for 6-month follow up (103.53±41.23) was statistically greater than in the digital technique (62.33±26.29) (p=0.005) No statistically significant difference was observed between groups during the 3-month follow-up (p=0.724) ( Table 1 , Figure 2 ).

**Table 1:** Evaluation of the Volumetric Loss of Occlusal Splints

		Conventional production method		Digital production method		p^1^
		Mean±SD	Median (Min-Max)	Mean±SD	Median (Min-Max)	
Volumetric loss	3rd month	52,27±37,19	38 (9-142)	43,93±23,24	50 (9-86)	0.724
	6th month	103,53±41,23	98 (25-168)	62,33±26,29	66 (13-97)	**0.005**
p^2^	**0.003**					

(p<0,05) (CI %95)

VAS scores mean values were evaluated for significant differences in Table 2 . There was no significant difference between the two types of splints across treatments.

**Table 2:** Evaluation of the VAS Scores

		Conventional production method		p^2^	Digital production method		p^2^	p^1^
		Mean±SD	Median (Min-Max)		Mean±SD	Median(Min-Max)		
VAS	Beginning	6,53±1,73	7 (2-9)	**<0.001**	6,87±1,36	7 (5-9)	**<0.001**	0.751
	1st month	5,47±1,81	5 (2-9)		5,87±1,55	6 (3-8)		0.473
	2nd month	4,4±1,72	5 (1-8)		4,27±1,79	4 (1-7)		0.800
	3rd month	2,87±1,25	3 (1-5)		3±1,81	3 (0-7)		1.000
	4th month	1,53±0,99	1 (0-3)		2±1,73	1 (0-6)		0.665
	5th month	0,87±0,92	1 (0-3)		1,13±1,3	1 (0-4)		0.741
	6th month	0,47±0,74	0 (0-2)		0,4±0,74	0 (0-2)		0.738
p^2^	0.751							

(p<0,05) (CI %95)

No serious adverse events were reported in either group. No cases of occlusal interference, mucosal irritation, or device fractures were observed.

## Discussion

Our null hypothesis was partially rejected. Conventionally manufactured splints showed significantly higher wear loss values compared with digitally manufactured ones. Additionally, VAS scores—which represent pain levels during treatment—presented no differences between the two groups.

Latest advancements in bruxism classification includes the STAB instrument introduced in 2023 and which provides a thorough, multidimensional tool to evaluate bruxism by looking at its status, possible effects, causes, and other existing conditions. Here we used only its Axis A, which consists of subject-based and clinically based assessments, for self-reported information and clinical evaluations.^5^ PSG, the gold standard for bruxism diagnosis, can only be performed in a sleep laboratory which is expensive for patients and cannot be applied chairside in the dental clinic. Thus, we preferred clinic-based diagnostic methods even if it represented a study limitation.

Several studies have sought to provide canine-guided occlusion on the surface of stabilization splints in bruxism patients and preventing the occlusion from changing due to usage-related attrition. Benli and Ozcan^7^ (2023) reported that occlusal splint therapy is accepted as a gold standard for treating bruxism. Wang, et al.^14^ (2020), Hirai, et al.^15^ (2017), and Iizumi, et al.^16^ (2023) stated that occlusal splint therapy should be applied to individuals with bruxism.

Numerous studies have assessed the effects of hard and soft occlusal splints on bruxism. Cruz-Reyes, et al.^17^ (2011) reported that hard stabilization splints have shown better effects on masseter muscle contraction than soft occlusal splints. Poorna, et al.^18^ (2022) found that hard occlusal splints exhibited earlier reduction effects on the symptoms of temporomandibular joint disorders than soft ones. Okeson^6^ (2019) reported that while hard splints reduced bruxism activity, soft splints did not. Based on these findings, we included hard occlusal splints into our present research.

Despite several *in vitro* studies examining the wear of splint materials, *in vivo* investigations on this issue are still scarce.^14 , 15 , 16^ Grymak, et al.^19^ (2022) observed an inadequate number of *in vivo* research concerning three-dimensional additive manufacturing, highlighting the need for *in vivo* studies on the wear properties of occlusal splints manufactured using this method. Following this observation, we chose three-dimensional additive manufacturing for comparison with conventional manufacturing processes.

Despite advantages, PMMA addition in conventional splint manufacturing is not regarded as the optimal material for occlusal splints due to prolonged fabrication time, allergenic components, unpleasant taste, dimensional instability, residual monomer release, fracture susceptibility, and undesirable shape or color.^20^

Advancement of digital technologies in dentistry has led to the evolution of computer-aided design and manufacturing that eliminate the disadvantages of traditional techniques by supporting both subtractive and additive splint manufacture. Additive manufacturing offers advantages over subtractive processes, including reduced material waste, lower energy consumption, and no requirement of manufacturing tools.^21^ Hence the use of additive manufacturing techniques in this research.

Conventional splint fabrication methods involve vacuum heat molding a polyethylene sheet created from refractory castings followed by occlusal customization using polymethylmethacrylate (PMMA), which can be produced via heat-polymerized, auto-polymerized, or light-polymerized acrylic resins.^22^

Benli, Al-Haj Husain and Ozcan^20^ (2023) reviewed occlusal splint materials manufactured under different methods and concluded that PMMA-based materials show superior flexural modulus, flexural strength, elastic modulus, and fracture toughness, regardless of their manufacturing processes. Thus, we used PMMA-based materials to examine their advantages and differences based on their fabrication method.

León Velastegui, et al.^23^ (2022) stated that no standardized procedure exists for wear loss measurement; however, Geomagic and Polywork applications emerge as the preferred techniques for imaging wear measurement. DeLong^24^ (2006) examined various techniques for assessing wear loss and concluded that 3D scanning is the most effective method for comparing surface images. Hirai, et al.^15^ (2017) conducted a wear assessment in occlusal splints, indicating that surface wear and deformations can be detected by digital scanning and comparison of two images together with their corresponding data.

Bruxism can cause headaches, neck pain, muscle spasms, and pain in the masticatory muscles. The VAS scale is a validated and reliable measure of pain intensity during rehabilitation.^25^ Studies have used VAS scales to assess pain intensity during treatment of temporomandibular disorders and bruxism.^8 , 26 , 27 , 28 , 29^ Hence our use of VAS scales to evaluate pain scores for two different splint manufacturing methods.

*In vitro* research on the wear loss of occlusal splints have utilized chewing simulators to simulate bruxism and have conducted material evaluations under laboratory conditions. Wesemann, et al.^9^ (2021) examined the wear rates of conventionally manufactured, CAD-CAM, and 3D printed materials used for occlusal splint fabrication against human enamel, concluding that conventional materials exhibited the least mean volume loss.

Lutz, et al.^30^ (2019) assessed the wear of conventionally manufactured and 3D printed materials against human enamel, finding that conventionally generated PMMA showed the least volumetric loss, but with no significant difference when compared with CAD-milled materials.

Reyes-Sevilla, et al.^31^ compared the wear rates of different occlusal splint materials against resin composite materials, showing that the wear outcomes of various PMMA materials differ based on their manufacturing processes. Conventionally made PMMA presents the highest wear rates, whereas printed PMMA had the lowest compared with milled PMMA.

Benli, et al.^10^ (2020) evaluated the wear rates of PMMA, EVA (ethylene- vinyl acetate), PC (polycarbonate) and PETG (polyethylene therephthalate glycol) materials after thermomechanical fatigue loading, concluding that PEEK materials have the lowest volume loss after 60.000 cyclic loading.

Such differences in the volumetric loss of conventional PMMA investigated *in vitro* studies could be due to material comparisons, variations in thermomechanical loading protocols, and attempts to simulate the oral environment with distilled water.

Despite numerous *in vitro* studies on the wear loss of splint materials, only a few *in vivo* studies focus this issue.

Wang, et al.^14^ (2020) assessed the wear volume loss and depth of occlusal surfaces for splints fabricated digitally from PEEK material and conventionally from PMMA material over a 12-week period in individuals with sleep bruxism. The authors reported that digitally produced PEEK-based splints had statistically lower volumetric loss values than conventionally manufactured ones. Studies highlight the need to observe long-term wear effects of splint use in people with sleep bruxism. In the literature, occlusal splint therapy is defined as a short-term treatment for the first 3 months of follow-up and long-term for treatments lasting more than 3 months. Kuzmanovic, et al.^32^ (2017) found no significant differences between patients who underwent 1 year and 6 months of long-term splint use compared with short-term treatment.

Wear loss reduces the therapeutic effects of occlusal splints by reducing protection and altering occlusal contacts. Moreover, the decrease in shock absorption leads to an increase in bruxism-related damages, whereas reduction in vertical dimension from wear loss increases muscle tension. Conversely, adjusting occlusal splint materials with high wear is simpler whereas adjusting wear-resistant material is time-consuming. Due to these characteristics, it is crucial to select the appropriate material for patients when manufacturing occlusal splints.^9 , 14 , 19^

Here, volumetric loss assessment of printed PMMA and conventional PMMA following six months of long-term clinical application showed no significant difference between groups after 3-month follow-up, but the printed PMMA exhibited significantly lower volumetric loss values after six months.

As for the effects of occlusal splints on pain levels, studies have used a visual analog scale (VAS) to assess it and reported that occlusal splints provide a pain-relieving effect when used regularly.^8 , 26 , 27 , 28 , 29^ Kaya and Ataoğlu^26^ (2021) found that occlusal splint treatment has significantly reducing effects on VAS scores. Gomes et al.^27^ (2015) compared the pain levels by VAS scores of individuals with bruxism and concluded that occlusal splint treatment significantly reduced them.

Our results showed that occlusal splints reduced pain levels in affected individuals, in line with previous research, but the manufacturing method had no effect on this outcome.

Study limitations include the use of self-reporting and positive clinical examination for bruxism diagnosis instead of PSG recording and the impossibility to standardize bruxism severity in the participants. Splints fabricated from conventional PMMA exhibited significantly higher volumetric loss than printed PMMA after six months of use. Regardless of the manufacturing method used, VAS scores among participants showed a significant decrease in both study groups with no significant difference between the two splint types regarding VAS scores during treatment. Further clinical studies with extended follow-up periods, larger sample size, and variety of materials are necessary on this topic.

## Conclusion

Occlusal splint wear can gradually alter the balance of occlusal contacts and potentially reduce its therapeutic effectiveness by disrupting even force distribution, altering guidance patterns, and increasing muscle activity. This deterioration can compromise the device's ability to manage bruxism and occlusal stability. Thus, selecting wear-resistant materials is crucial for maintaining long-term clinical outcomes, ensuring consistent occlusal support and preserving the intended therapeutic benefits.

Data availability

The datasets generated during and/or analyzed during the current study are available from the corresponding author on reasonable request.
